# Internet and Free Press Are Associated with Reduced Lags in Global Outbreak Reporting

**DOI:** 10.1371/currents.outbreaks.cecdec16fa17091eea4c4a725dba9e16

**Published:** 2014-10-30

**Authors:** Lindsey McAlarnen, Katherine Smith, John S. Brownstein, Christopher Jerde

**Affiliations:** Florida State University College of Medicine, Tallahassee, Florida, USA; Department of Ecology and Evolutionary Biology, Brown University, Providence, Rhode Island, USA; Boston Children’s Hospital, Harvard Medical School, Boston, Massachusetts, USA; Environmental Change Initiative & Dept. of Biological, University of Notre Dame, Notre Dame, Indiana, USA

## Abstract

Background: Global outbreak detection and reporting have generally improved for a variety of infectious diseases and geographic regions in recent decades. Nevertheless, lags in outbreak reporting remain a threat to the global human health and economy. In the time between first occurrence of a novel disease incident and public notification of an outbreak, infected individuals have a greater possibility of traveling and spreading the pathogen to other nations. Shortening outbreak reporting lags has the potential to improve global health by preventing local outbreaks from escalating into global epidemics. Methods: Reporting lags between the first record and the first public report of an event were calculated for 318 outbreaks occurring 1996-2009. The influence of freedom of the press, Internet usage, per capita health expenditure, and cell phone subscriptions, on the timeliness of outbreak reporting was evaluated. Results: Freer presses and increasing Internet usage correlate with reduced time between the first record of an outbreak and the public report. Increasing Internet usage reduced the expected reporting lag from more than one month in nations without Internet users to one day in those where 75 of 100 people use the Internet. Conclusion: Advances in technology and the emergence of more open and free governments are associated with to improved global infectious disease surveillance.

## Introduction

The increasing number of pathogens that emerge locally but quickly spread through global trade and travel networks, most recently avian influenza A ( H7N9), Middle East respiratory syndrome coronavirus (MERS-CoV) and Ebola virus disease, illustrate the need for smarter infectious disease surveillance^1^^,^ 2 . Global outbreak detection and reporting have generally improved for a variety of diseases and geographic regions in recent decades^3^ . Nevertheless, lags in outbreak reporting remain a threat to global public health and security, and it is unclear what factors decrease their length, although improved communications and implementation of the International Health Regulations (IHR) have been implicated.

The 2003 Severe Acute Respiratory Syndrome (SARS) epidemic illustrates the global public health risks associated with reporting lags. On 11 February 2003, the Chinese Ministry of Health in Beijing reported 300 cases and 5 deaths of SARS to the World Health Organization (WHO), later acknowledging the outbreak dated back to November 16, 2002^4^ . In late February a physician infected with SARS traveled from the epidemic origin in Guangzhou to Hong Kong, infecting residents and international travelers^5^ . SARS spread to 29 countries, infected >8000 people in 8 months and is estimated to have cost the global economy 30-100 billion USD^6^ . Early detection and isolation along with transparent communication with the public regarding the true disease magnitude could have prevented SARS from escalating into an international epidemic^5^ .

Early disease detection and patient isolation, along with more rapid and transparent communication with the public regarding the true disease magnitude, could prevent emergence of some diseases, such as SARS, from escalating into an international epidemic^4^^,^ 5. Shortening outbreak reporting lags has the potential to save lives, strengthen national security, and safeguard the global economy^6^. We compiled reporting data and potential explanatory factors for 318 human infectious disease outbreaks that occurred at various locations around the world during the period 1996-2009^3^ . We tested the hypothesis that increased freedom of the press, health expenditure and communications infrastructure (specifically Internet usage and cell phone subscriptions), contribute to decreased reporting lags.

## Methods

Infectious disease outbreaks reported by Chan et al. (2010) that were selected for analyses included those that could be linked to a specific causal pathogen (to the species, genus, or family level). We eliminated 52 reports of acute or undiagnosed symptoms and diseases from the initial dataset of 398 outbreaks and also removed the 2005 Chikungunya outbreak on Reunion Island because France’s social and demographic attributes did not accurately represent the overseas department. Additionally, 27 outbreak reports were eliminated because explanatory factors for the country-year of occurrence were not available.

A free press contributes to governmental transparency, providing citizens with information and promoting justice^7^ . The freedom of the press index is a numerical value between 0 and 100, where 0 is the freest, assigned to each of 196 countries on the basis of 23 methodology questions and 109 indicators of the legal environment, the political environment, and the economic environment. Countries with a total score between 0 and 30 are designated ‘free’; countries with scores between 31 and 60 are ‘partly free’; and countries with scores 61-100 are considered ‘not free’^8^ .

Disease control efforts reduce pathogen prevalence^9^ . A country’s health expenditureis the sum of public and private health expenditures as a ratio of the total population. This represents the most recent total health expenditure purchasing power parity (PPP) conversion in the international dollar, allowing us to assess each country’s health expenditure with a common currency^10^ . The PPP conversation protects against misleading inferences due to change rate differences between global currencies.

The Internet is used to collect and disseminate information, promote governmental transparency, and is often the identified as one of many vital communication components needed for a robust public health surveillance program^11^^,^ 12 . Internet usage is the number of people with access to the World Wide Web per 100 people 13 .

Cell phones can connect rural areas to developed health centers and can also serve as infectious disease reporting devices when equipped with mobile Internet capabilities^14^ . We evaluate cell phone subscriptions using mobile cellular subscriptions per 100 people, which are either prepaid or post-paid subscriptions to a public cellular mobile telephone service^15^ . The number of cellular phone subscriptions per 100 people can be a number over 100 when individuals have more than 1 subscription.

Internet coverage and cell phone subscriptions are representative of two communications infrastructure advancements that globally occurred during the study’s duration. Previous communication advancements, such as Telex likely had similar impacts when first implemented 23 ^,^ 24 .

Zoonotic pathogens are infectious agents that develop, mature, and reproduce in non-human hosts, but have the potential to spill over and infect humans (e.g. Nipah virus, rabies), and human specific pathogens are those entirely restricted to the human population (e.g. measles)^16^ . Human specific pathogens are more globally distributed than zoonotic pathogens, which are far more localized in their geography^16^ . Familiarity with outbreaks of different host types may affect the timeliness with which outbreaks are reported. For example, a common human specific pathogen may be diagnosed more efficiently than a rare or novel zoonotic pathogen. We evaluated host type (human specific pathogens vs. zoonotic pathogens) as a possible explanatory variable.

Outbreak reporting lags were previously calculated as the time from the first record of the onset of the event to the first public report^3^ . We used the discrete waiting time formulation captured by the geometric distribution, Pr(Y=*y*)=*p*(1-*p*)*y*, where *y*, the observed lag, is measured in days (0, 1, 2,…) and *p* is the daily probability the outbreak is reported (0≤*p*≤1). To include covariates into the formulation, the probability is estimated under a logit transformation such that,

\begin{equation*}p=\frac{e^{\beta_{0}+\beta_{1}(x_{1})+ . . .} }{1+e^{\beta_{0}+\beta_{1}(x_{1})+ . . .}} \end{equation*}

where *β’*s are a set of linear parameters and *x*’s are the variables of interest (e.g. freedom of the press index). With five independent factors, there are 32 models tested. We used Akaike’s Information Criterion (AIC) for the purposes of model selection^17^ .

## Results

The best model included four covariates (Table 1). However, freedom of the press index and Internet usage were most strongly associated with reduced lags in outbreak reporting. Countries with open media policies and a large proportion of Internet users are fastest to report outbreaks (Fig. 1). The association between increasing reporting lags with increasing cell phone subscriptions and health expenditure is a weak but unintuitive relationship. In large part, this outcome appears driven by the relatively large cell phone coverage in outbreak prone, developing countries such as Vietnam, Mexico, and China, and outbreaks occurring in developed countries with relatively large health expenditure (e.g. a 2001 legionellosis outbreak in Norway and repeated outbreaks of West Nile Virus in the United States).

**Cumulative probability of an outbreak being reported to the public based on a country’s freedom of the press index.**
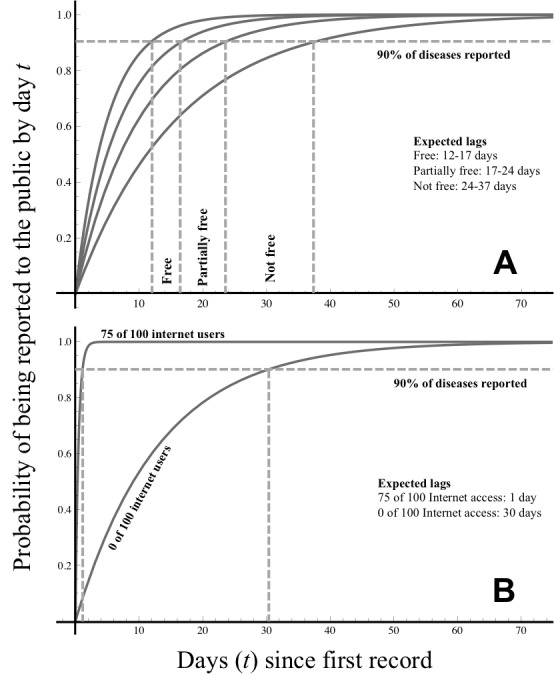
Cumulative probability of an outbreak being reported to the public based on a country’s freedom of the press index (A), where the upper and lower limits of free, partly free, and not free (0, 30, 60, 100) are shown with gray boundary lines. Internet usage (B) of 75/100 Internet users and 0/100, the upper and lower bounds of observed global usage, are delineated with gray lines.

**Table 1. Modeling results for lags between first record and public report.** Variable abbreviations: Freedom of the press index (FP), Health expenditure (HE), Internet usage (I), host type (H), Cell phone subscriptions (Cell). Best model under AIC selection procedure; ΔAIC < 2 no evidence for model differentiation; 2<ΔAIC<8 is week evidence for model differentiation; ΔAIC>8 strong evidence for differentiation

**Model**	**Variables**	***k***	**log-likelihood**	**AIC**	**ΔAIC**	***w***
0	Constant only	1	-1093.43	2188.87	25.96	<0.0001
1	FP	2	-1087.30	2178.60	15.69	0.0002
2	HCE	2	-1090.38	2184.76	21.85	<0.0001
3	I	2	-1085.52	2175.05	12.14	0.0012
4	H	2	-1093.23	2190.45	27.55	<0.0001
5	Cell	2	-1092.11	2188.21	25.31	<0.0001
6	FP, HCE	3	-1086.82	2179.63	16.73	0.0001
7	FP, I	3	-1082.08	2170.16	7.26	0.0137
8	FP, H	3	-1087.17	2180.33	17.43	<0.0001
9	HCE, Cell	3	-1086.84	2179.68	16.78	0.0001
10	HCE, I	3	-1085.16	2176.32	13.41	0.0006
11	HCE, H	3	-1090.08	2186.15	23.25	<0.0001
12	HCE, Cell	3	-1090.31	2186.62	23.71	<0.0001
13	I, H	3	-1084.90	2175.79	12.89	0.0008
14	I, Cell	3	-1083.76	2173.52	10.61	0.0026
15	H, Cell	3	-1091.59	2189.18	26.28	<0.0001
16	FP, HCE, I	4	-1078.97	2165.94	3.03	0.1136
17	FP, HCE, H	4	-1086.63	2181.27	18.37	0.0005
18	FP, HCE, Cell	4	-1086.69	2181.38	18.47	0.0005
19	FP, I, H	4	-1081.65	2171.29	8.39	0.0078
20	FP, I, Cell	4	-1080.15	2168.31	5.40	0.0347
21	FP, H, Cell	4	-1086.57	2181.15	18.24	<0.0001
22	HCE, I, H	4	-1084.48	2176.96	14.06	0.0004
23	HCE, I, Cell	4	-1084.84	2177.69	14.79	0.0003
24	HCE, H, Cell	4	-1089.93	2187.86	24.95	<0.0001
25	I, H, Cell	4	-1083.39	2174.78	11.87	0.0001
26	FP, HCE, I, H	5	-1078.64	2167.28	4.37	0.0582
27	FP, HCE, I, Cell	5	-1076.45	2162.90	0	0.517
28	FP, I, H, Cell	5	-1079.93	2169.86	6.96	0.0159
29	HCE, I, H, Cell	5	-1082.77	2175.54	12.63	0.0009
30	FP, HCE, H, Cell	5	-1086.44	2182.87	19.97	<0.0001
31	FP, HCE, I, H, Cell	6	-1076.26	2164.52	1.62	0.2299

The shortest expected reporting lags occurred in the Americas, Europe, and Australia whereas the longest expected lags occurred in central and northern Africa and central Asia (Fig.2). The expected lag between first record and public report decreased over time in several countries. China’s expected reporting lag decreased from 13.3 days in 2003 to 5.7 days in 2009, possibly a result of scrutiny in the aftermath of SARS^5^ (Fig. 3) and the initiated implementation of IHR. An increase in press freedom and Internet usage in the Democratic Republic of the Congo was associated with a drop in the expected reporting lag to 12.0 days in 2007. However, the expected lag rose to 17.6 days the following year, perhaps due to a government transition in which a new constitution was adopted and a single party no longer controlled the media^18^ (Fig. 3).

**Global risk map of expected lags between first record and public report (2009).**
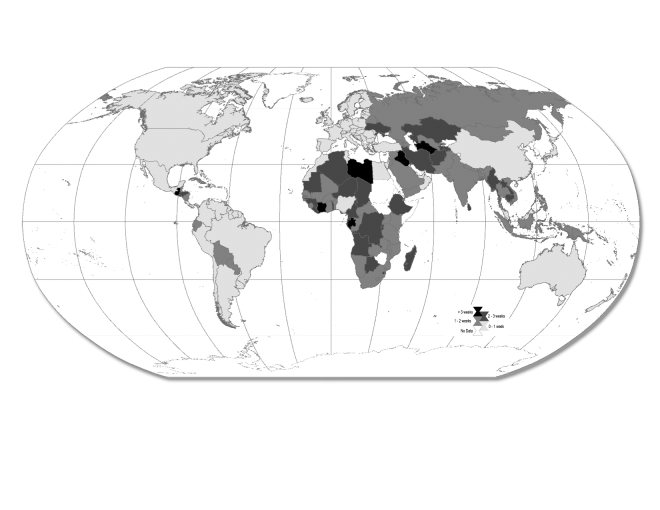
Light gray indicates countries with expected lags less than one week and countries in black have expected lags of four weeks or more.

**Expected waiting time (in days) between first record and public report.**
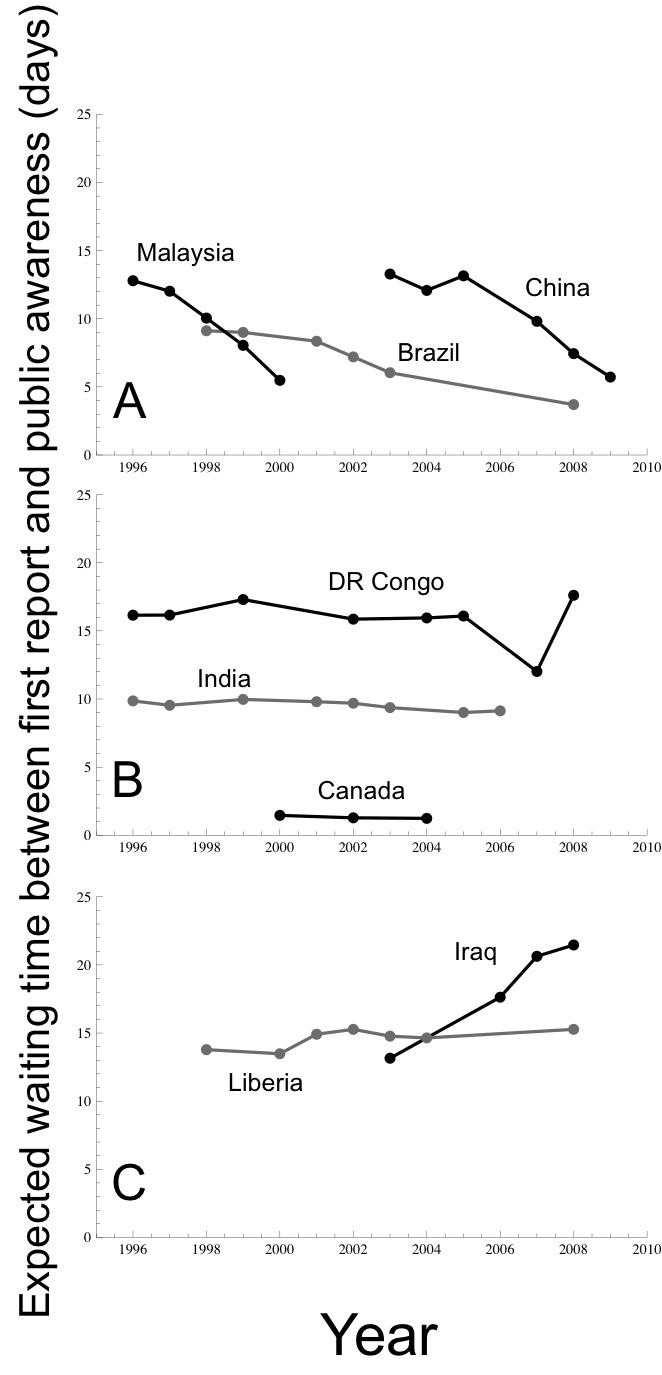
Many countries expected waiting times decreased over time (A), remained relatively constant (B), and a few increased (C).

## Conclusion

The Internet’s ability to facilitate syndromic surveillance^19^ , communicate eyewitness accounts^11^, and provide unofficial reports that supplement traditional public health approaches to monitoring evolving events^20^^,^ 21 reveals a unique role the general population can play in outbreak risk reduction^22^ *.* Health officials are quickly becoming aware of the value of publicly generated digital disease surveillance.

In places with regulated Internet and controlled press, there is the potential for misleading propaganda that obfuscates public reporting of emerging diseases. This interplay between the ability to communicate with new technology and the regulation of technology and information will almost certainly be dynamic, and Internet based surveillance efforts will need to adapt to changing social network platform preferences and fluctuations in government censorship of the various social network platforms. Careful attention should be paid to identify countries where the Internet could provide sufficient disinformation as to hinder reporting of emerging diseases.

The early stages of the MERS-CoV outbreak were first reported by ProMED-mail and the bioinformatics community is relying on digital surveillance to detect signals of H7N9 in the next influenza season^22^ . However, complete reliance on technology may be a dangerous pathway for disease detections and predictions, particularly if they are not reevaluated and adjusted as better data and improved techniques develop^25^ . Nevertheless speed and cost effectiveness of social media, in combination with growing user engagement and advances in machine learning hold great promise for even better digital disease detection. In light of advances in technology and the emergence of more open and free governments, an era of advanced global infectious disease surveillance may be upon us.

## Competing Interests

The authors have declared that no competing interests exist.
